# Fabrication of Convex PDMS–Parylene Microstructures for Conformal Contact of Planar Micro-Electrode Array

**DOI:** 10.3390/polym11091436

**Published:** 2019-09-02

**Authors:** Woo Ram Lee, Changkyun Im, Hae-Yong Park, Jong-Mo Seo, Jun-Min Kim

**Affiliations:** 1 Dental Life Science Research Institute, Seoul National University Dental Hospital, Seoul 03080, Korea; 2 Department of Electrical and Computer Engineering, and Institute of Engineering, Seoul National University, Seoul 08826, Korea; 3 Inter-university Semiconductor Research Center, Seoul National University, Seoul 08826, Korea; 4 Dental Research Institute, Seoul National University School of Dentistry, Seoul 03080, Korea; 5 Department of Physiology, College of Medicine, Hallym University, Chuncheon 24252, Korea; 6 Department of Electronic Communication Engineering, Gyeonggi University of Science Technology, Siheung 15073, Korea

**Keywords:** convex microstructure, hybrid microstructure, molding fabrication process, micro-electrode array (MEA), conformal contact, polymer, electrocorticography (ECoG), rat olfactory bulb, beta frequency band, respiration

## Abstract

Polymer-based micro-electrode arrays (MEAs) are gaining attention as an essential technology to understand brain connectivity and function in the field of neuroscience. However, polymer based MEAs may have several challenges such as difficulty in performing the etching process, difficulty of micro-pattern generation through the photolithography process, weak metal adhesion due to low surface energy, and air pocket entrapment over the electrode site. In order to compensate for the challenges, this paper proposes a novel MEA fabrication process that is performed sequentially with (1) silicon mold preparation; (2) PDMS replica molding, and (3) metal patterning and parylene insulation. The MEA fabricated through this process possesses four arms with electrode sites on the convex microstructures protruding about 20 μm from the outermost layer surface. The validity of the convex microstructure implementation is demonstrated through theoretical background. The electrochemical impedance magnitude is 204.4 ± 68.1 kΩ at 1 kHz. The feasibility of the MEA with convex microstructures was confirmed by identifying the oscillation in the beta frequency band (13–30 Hz) in the electrocorticography signal of a rat olfactory bulb during respiration. These results suggest that the MEA with convex microstructures is promising for applying to various neural recording and stimulation studies.

## 1. Introduction

The neural electrode is an important element in understanding the electrochemical mechanisms for various neurological disorders such as Parkinson′s disease, epilepsy, spinal cord injury, stroke, and sensory deficits [1]. The electrodes are categorized into non-invasive and implantable electrodes depending on the location of the electrode site [2]. The non-invasive electrode is widely used in clinical and psychiatric fields because it can measure electrical signals from the brain by being placed on the scalp without surgical intervention. However, since the electrical signals have poor spatial resolution and signal-to-noise ratio, and it is difficult to determine where they occur in the brain, multiple electrode placement on the scalp is required [3]. The implantable electrode is an electrical conductor that is placed on the surface of the cortex or into the brain to record or modulate neural signals. Although surgical intervention is required for electrode placement, the clinical use of neuro-stimulation with the implantable electrode has been approved for the symptomatic relief of epilepsy and depression, and for the treatment of Parkinson’s disease, because it may avoid many adverse effects associated with medications [4].

Electrocorticography (ECoG), which is performed to localize the epileptic foci and to assess the function of the cerebral cortex, is a representative type of neural recording technique using epidurally or subdurally placed implantable planar micro-electrode arrays (MEAs) [5].

The MEAs are also used as a conduit to deliver therapeutic cortical stimulation for patients with epilepsy, neuropathic pain disorders, movement disorders, and psychiatric disorders [6]. There are several requirements for materials used in the fabrication of the MEAs: (1) biocompatibility; (2) high moisture resistance to prevent cross-talk and electrode corrosion; (3) high flexibility to adhere tightly to the curved surface of the brain and (4) low stiffness to reduce inflammatory response [7,8,9,10,11]. Polymeric materials such as benzocyclobutene (BCB), parylene-C, polydimethylsiloxane (PDMS), polyimide (PI), polynorbornene (PNB), SU-8, and liquid crystal polymer (LCP) are known to meet the requirements [12]. Among them, PDMS is the most attractive material due to its properties including biocompatibility, flexibility, chemical inertness, and low Young′s modulus [13,14]. In particular, Sylgard 184, one of the PDMSs, is widely used as a coating material in implantable devices with lower Young′s modulus than other polymers. However, it is difficult to deposit the metal pattern on the surface of PDMS due to the poor adhesion that results from the different degree of thermal expansion between the metal and the PDMS layers [15,16,17,18]. Both parylene-C and PI have excellent metal adhesion, but having a small thickness makes handling and manufacturing difficult [19]. In fact, to date, there is no single material that meets all the requirements. As a way to overcome this problem, Ochoa and colleagues [20] introduced a PDMS–Parylene hybrid layer combining the advantages of both materials. The hybrid layer shows a desirable combination of stiffness and flexibility while maintaining an uncomplicated fabrication process and metal adhesion.

**Figure 1:**
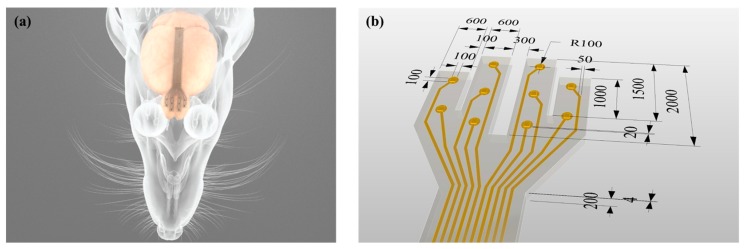
Schematic of the micro-electrode arrays (MEA) with ten convex electrode sites in four arms for in vivo Electrocorticography (ECoG) recording from rat olfactory bulb. (**a**) Experimental setup. (**b**) The overall dimensions of the MEA. The unit of length is micrometer (μm).

The most important property to consider when using a polymeric material as a substrate for an MEA is surface hydrophobicity [21]. Due to this property, it is known that air pocket entrapment can occur depending on the aspect ratio and contact angle of the microstructure comprising a polymeric material [22,23,24]. In particular, the air pocket trapped above the electrode site interferes with the close contact between the MEA and the curved surface of the cortical surface and reduces signal quality [15]. Therefore, in this paper, we report on a novel method of fabricating a planar MEA capable of measuring a neural signal while maintaining conformal contact between its electrode site and the cortical surface. The MEA is made of PDMS and Parylene-C, which are representative polymers, thus having high biocompatibility, moisture resistance, and flexibility. Further, based on the theoretical background of the occurrence of the air pocket entrapment, which is a problem of the conventional MEAs having concave microstructures, the validity of the convex microstructure implementation is demonstrated. Finally, the feasibility of the MEA is proved by recording the ECoG signal from the rat olfactory bulb (Figure 1a).

## 2. Materials and Methods 

Figure 1b shows an MEA designed and fabricated to form convex microstructures through a molding fabrication process. The MEA consists of parylene, metal and PDMS layers, and the thickness from the bottom to the surface is about 204 μm. The MEA has four arms with a length of 1.0–2.0 mm and a width of 600 μm. On each arm, there are two or three electrode sites with dimensions of 100 μm × 100 μm protruding about 20 μm from the outermost layer surface.

### 2.1. Fabrication

**Figure 2:**
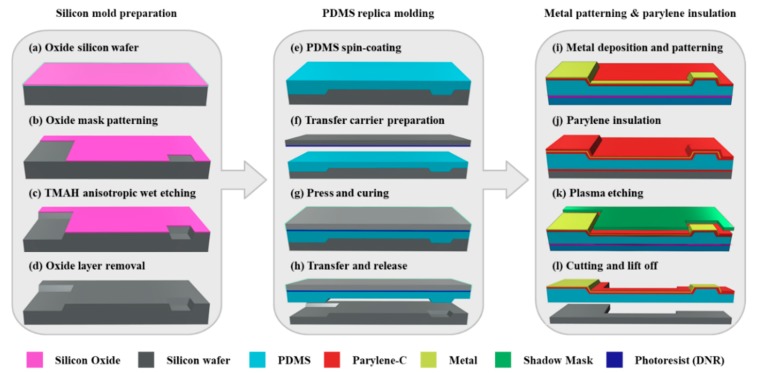
Schematic of the fabrication process flow for an MEA with replicated convex microstructures.

The fabrication process of the MEA with convex microstructures is divided into three parts: (1) silicon mold preparation; (2) PDMS replica molding, and (3) metal patterning and parylene insulation. The entire fabrication process is schematized in Figure 2.

#### 2.1.1. Silicon Mold Preparation

A silicon wafer having a crystal orientation of (100) with an oxide film (SiO_2_) of 300 nm thickness is used as a silicon mold (Figure 2a). First, negative photoresist (DNR-L300, DONGIN, Seoul, Korea) is spun onto the surface of the silicon wafer at 1300 rpm, and then the photolithography is carried out using mask aligner (MA-6, KARL_SUSS, Garching, Germany) with the energy of 360 mJ/cm^2^. A SiO_2_ dry etching is conducted by using a magnetically enhanced reactive ion etcher (MERIE, Applied Materials P-5000). The dry etching process is performed for 1 min under CHF_3_ gas flow rate of 25 sccm, CF_4_ gas flow rate of 5 sccm, Ar gas flow rate of 50 sccm, a pressure of 130 mTorr and a power of 600 W. After the completion of the dry etching, the photoresist is removed by acetone (Figure 2d). Since the silicon wafer has a cubic crystal structure, anisotropic etching is possible due to the difference in etching rate depending on the crystal orientation. The silicon wafer is immersed in 25 wt % tetramethylammonium hydroxide (TMAH) alkaline solution at 80 °C for the anisotropic wet etching. Since the etching rate is 0.5 μm/min, the etching condition is maintained for 40 min to obtain concave structures with a depth of about 20 μm (Figure 2c). Concave structures having a sidewall slope angle of 54.7° can be achieved by using the silicon wafer with a crystal orientation (100). After the anisotropic wet etching, the silicon wafer is immersed in 5% hydrofluoric acid at 25 °C for 1 min to remove the oxide layer and then rinsed several times with deionized water to form the silicon mold (Figure 2d).

#### 2.1.2. PDMS Replica Molding

A PDMS layer with convex structures is prepared by pouring and spin-coating (500 rpm for 30 s) a mixture of base and curing agent (Sylgard 184 kit, Dow corning, Midland, USA) at a ratio of 10:1 (*w*/*w*) onto the prefabricated silicon mold (Figure 2e). Prior to pouring the PDMS, the silicon mold is first silanized with a hydrophobic dodecyltrichlorosilane (DTS, Sigma-Aldrich, St. Louis, USA) for 2 h under 1 bar vacuum pressure to facilitate subsequent release of the PDMS. The other silicon wafer as a transfer carrier is spin-coated with a negative photoresist (DNR-L300, Dongjin Semichem, Seoul, Korea) (Figure 2f), and then superimposed on the surface of the PDMS with a weight of 20 kg. The PDMS is cured at 60 °C in the vacuum oven chamber for 5 h (Figure 2g). The adhesion force between the PDMS and the photoresist is stronger than the adhesion force between the PDMS and the silane, so that the PDMS layer attached to the transfer carrier is separated from the silicon mold (Figure 2h).

#### 2.1.3. Metal Patterning and Parylene Insulation

For a stable gold micro-patterning, the surface of the PDMS layer is treated with oxygen plasma for 30 s under an O_2_ gas flow rate of 20 sccm, a pressure of 100 mTorr, and RF source power of 50 W, and then is coated with parylene. The surface of the parylene is also treated with the same oxygen plasma treatment to increase the surface energy, and sequentially deposited with titanium/gold (20 nm/200 nm) using an electron gun evaporator (ZZS550, MAESTEK, Mumbai, India). Then, it is spin-coated with the negative photoresist at 2500 rpm for 30 s and exposed at 360 mJ/cm^2^ using the mask aligner. The gold layer is patterned by wet-etching using an iodine–potassium-iodide solution (40:4:1 = DI water:I_2_:KI) at room temperature, and the titanium layer is plasma etched for 3 min under an SF6 gas flow rate of 30 sccm and a power of 100 W (Figure 2i). Parylene coating with a thickness of 2 μm is carried out on the entire area except for the contact pads (Figure 2j). In order to open the electrode sites, the parylene layer is covered with a shadow mask and then patterned through reactive ion etching (RIE) under an etching power of 100 W and an O_2_ gas flow rate of 50 sccm for 3 min (Figure 2k). The final product can be obtained by cutting and by dissolving the photoresist sacrificial layer in dimethyl sulfoxide (Figure 2l).

### 2.2. Mechanical and Electrochemical Characterizations

Since the polymer has a low surface energy, metal film deposited on the polymer surface can be easily separated. In order to evaluate the adhesion of titanium/gold (20 nm/200 nm) thin films on the PDMS and parylene substrates, peel tests were performed using Scotch^®^ tape (810, 3M, Maplewood, USA) after plasma treatment and metal patterning.

The electrochemical impedance of electrode sites was characterized in a phosphate-buffered saline solution using an impedance analyzer (IM6e, Zahner-Elektrik, Kronach, Germany) with an Ag/AgCl reference electrode and a Pt counter electrode. The impedance was measured in a wide frequency range from 1 Hz to 100 kHz.

### 2.3. Air Pocket Entrapment

It has been reported that air pocket entrapment can occur on the concave microstructure of the MEA due to the surface hydrophobicity [14]. In order to confirm the air pocket entrapment, five types of concave PDMS microstructures were fabricated with a width of 10 μm and depths of 2, 7, 10, and 32 μm, respectively, and placed into water at 25 °C with a contact angle of 45°.

### 2.4. Animal Experiments

Animal care protocol and experimental procedures were approved by the Institutional Animal Care and Use Committee of Hallym University (Protocol number Hallym 2016-49, Chuncheon, Korea). All experiments were carried out at the Department of Physiology, Hallym University, College of Medicine in accordance with relevant guidelines and regulations. Adult male Sprague-Dawley rats (300–350 g, n = 4) were used in this study. They were housed in a room with a 12 h light/dark cycle, and allowed access to food and water ad libitum. The rats were anesthetized with Zoletil 50^®^ (25 mg/0.5 mL/kg, Virbac) and Rompun (xylazine, 10 mg/0.5 mL/kg, Bayer AG, Leverkusen, Germany) by intraperitoneal injection, and supplemental doses were delivered intramuscularly when necessary.

Each animal was immobilized in a stereotaxic apparatus, and body temperature was maintained using a rectal probe and an electric heating blanket. After hair shaving and skin disinfection, the main olfactory bulb was exposed by a craniotomy. The MEA fabricated in this study was placed on the dura mater above the main olfactory bulb, and then ECoG signals were recorded for 60 s at a bandwidth of 0.3 to 300 Hz and a sampling rate of 1 kHz during respiration with odor-free air. The recording was repeated 20 times at 10 min intervals. The odor-free air was produced by filtering via a multi-filter that consists of silica gel layers and charcoal layers. The stream of the split air was humidified by passing through a distilled water container and delivered constantly (500 mL/min).

### 2.5. ECoG Analysis

ECoG analysis was performed offline in the Matlab software. The ECoG signals were down-sampled at 256 Hz in order to increase the computation efficiency, and then segmented into epochs of 4 s. The time-frequency representation was generated by the Morlet wavelet transform, and the averaged power spectral density (PSD) was derived using Welch’s modified method of spectral estimation.

## 3. Results and Discussion

**Figure 3:**
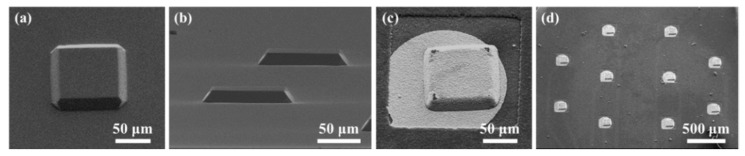
SEM images of fabricated convex structures. (**a**) Top view of a PDMS convex structure. (**b**) Side view of the PDMS convex structures. (**c**) Top view of an electrode site having a PDMS/parylene/metal hybrid convex structure. (**d**) Top view with different magnification of the electrode sites. Some images were adapted and modified from a doctoral dissertation [25,26].

**Figure 4:**
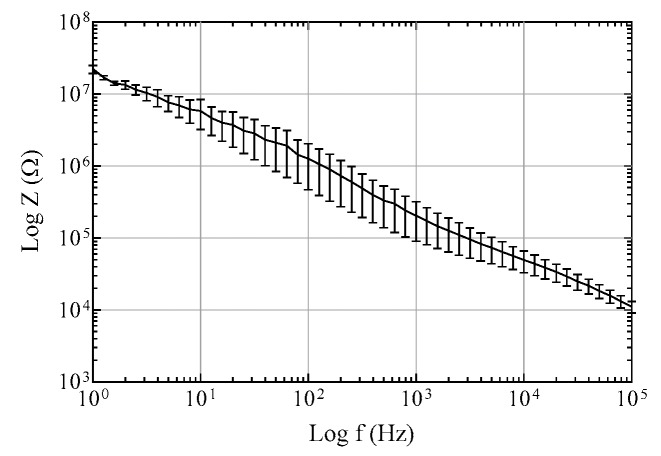
Impedance spectra of electrode sites with a diameter of 100 μm in a phosphate-buffered saline solution. The error bars are standard error of mean.

In this study, we propose a method to fabricate a flexible MEA with convex microstructures to facilitate conformal contact. The fabrication process includes the PDMS replica molding process to form convex microstructures that facilitate conformal contact, and the parylene-C coating to maintain low Young′s modulus and overcome the low metal adhesion of PDMS. Figure 3 shows photographs of the convex PDMS microstructures formed through the PDMS replica molding process, and the electrode sites laminated with two 2 μm-parylene layers and a metal layer. The dimensions of the convexly protruded electrode sites are approximately 100 μm × 100 μm × 20 μm. Figure 4 shows electrochemical impedance spectra in a wide frequency range from 1 Hz to 100 kHz. The measured impedance magnitude is 204.4 ± 68.1 kΩ at 1 kHz. We also showed that the MEA can be used as an ECoG electrode by recording neural signals from the rat olfactory bulb.

### 3.1. Fabrication of Hybrid PDMS/Parylene/Metal Layer

**Figure 5:**
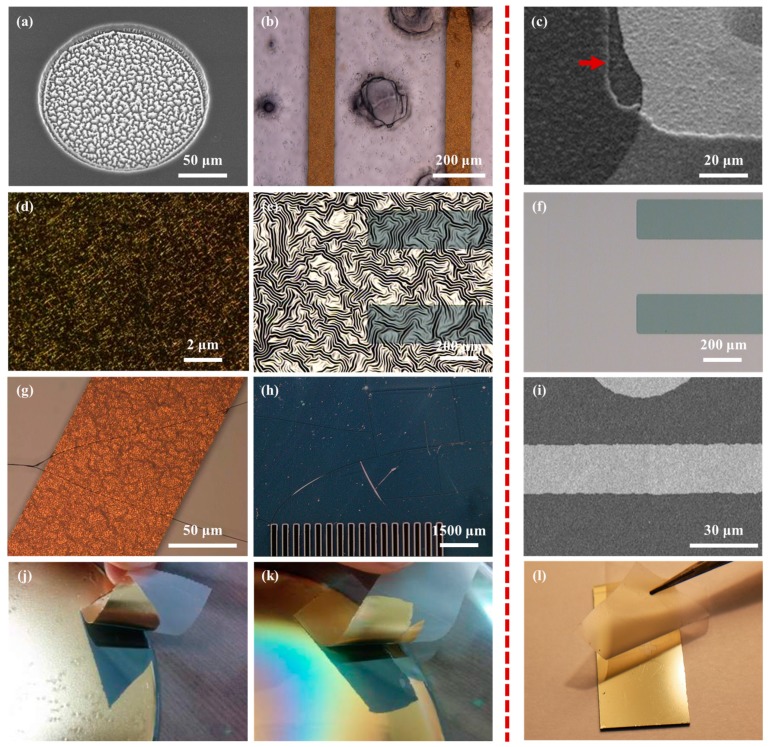
Comparison of fabrication process results with PDMS layer (the left of the red dotted line) and PDMS–parylene hybrid layer (the right of the red dotted line). (**a**) PDMS residues after dry etching; (**b**) PDMS dissolution; (**c**) Well-patterned parylene layer after dry etching. The red arrow shows the edge of etched parylene layer; (**d**) The surface of metal deposited on the PDMS surface damaged by excessive oxygen plasma treatment; (**e**) Metal film wrinkles by thermal expansion on PDMS layer; (**f**) Metal layer on PDMS–parylene hybrid layer after thermal treatment; (**g**) Cracks of the metal pattern and the photoresist layer due to the mismatch of the coefficient of thermal expansion; (**h**) Cracks of photoresist film on PDMS layer by thermal expansion; (**i**) Well-patterned metal layer on PDMS–parylene hybrid layer; (**j**) Exfoliation of metal layer deposited on the non- plasma- treated PDMS surface and (**k**) on the plasma-treated PDMS surface; (**l**) Preservation of metal layer deposited on the plasma-treated parylene surface on the PDMS layer. Some images were adapted and modified from a doctoral dissertation [25,26].

PDMS is one of the most commonly used elastomeric polymers for the fabrication of neural electrodes because it possesses ideal properties such as flexibility, biocompatibility, chemical stability, low cost, and durability [27,28,29]. However, there are three fundamental challenges in the process of fabricating an MEA using PDMS as shown in Figure 5. One is that it is difficult to carry out the etching process of the PDMS layer. Typically, dry or wet etching is used for micro-patterning and electrode site opening, but in the case of dry etching, low etch rate and PDMS residue generation are problematic, and in the case of wet etching, PDMS residue generation and PDMS swelling are problematic [30,31,32]. Another is that PDMS has a high coefficient of thermal expansion, making it difficult to produce micro-patterns through a standard photolithography process. Since the baking step of photolithography uses heat, thermal expansion is inevitable. Therefore, the mismatch of the coefficient of thermal expansion may cause damage to the metal pattern and photoresist layer as well as the PDMS structure [30]. The other challenge is that PDMS has weak adhesion to metals due to a low surface energy of 19–21 mJ/m^2^ [33]. For stable metal deposition, a process using a chemical treatment or a sacrificial layer is performed, but this process is complicated and costly.

Unlike the etching processes, the PDMS replica molding process can not only avoid the formation of residues, but also can quickly produce microstructures. Furthermore, since the mold can be reused, the production process can be simplified [34]. The use of the PDMS replica molding process can avoid the use of the PDMS etching process. However, the problems of weak metal adhesion due to low surface energy and PDMS thermal expansion due to the baking step of the photolithography still remain unresolved. It has been demonstrated that stable metal deposition is possible by improving the PDMS surface energy through plasma [35], 3-aminopropyltriethoxysilane (APTES) [36], 3-mercaptopropyltrimethoxysilane (MPTMS) [37], and 3-isocyanatopropyltriethoxysilane (IPTES) [38], but structural damage due to thermal expansion cannot be prevented [39].

As a way to overcome the problems caused by thermal expansion and low surface energy of PDMS, we noticed a hybrid structure in which parylene, PDMS, and metal layers are combined [20]. Parylene is known to be superior to PDMS on adhesion to metals [19] and to be easily patterned without residues using dry etching, as well as avoiding damaging the metal patterns due to heat or pressure by using a shadow mask during etching.

**Figure 6:**
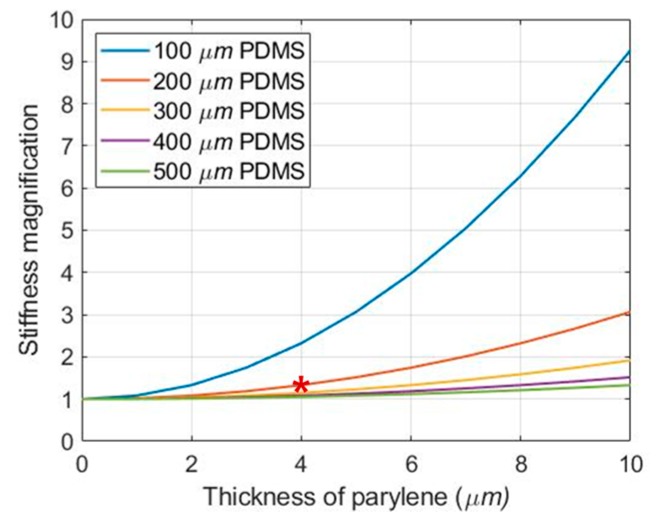
Magnification of the stiffness according to the thickness of parylene. The stiffness magnification was obtained by dividing the total stiffness by the stiffness of each PDMS ($D_{\mathrm{tot}}{/D}_{\mathrm{PDMS}}$). The red asterisk refers to the thickness of PDMS and parylene layer used in this paper.

Since the Young′s modulus of parylene is higher than that of PDMS, the thickness of the parylene layer affects the stiffness of the MEA. This means that one of the advantages of PDMS is lost. Therefore, in this study, we derived the effect of the 2 μm-parylene layer on the stiffness of the MEA through mathematical modeling [40]. The stiffness equation is as follows:

(1) $$D_{\mathrm{tot}}{\;=\;D}_{\mathrm{PDMS}}{\;+\;D}_{\mathrm{Parylene}}\;=\;\frac{Y_{\mathrm{PDMS}}h_{\mathrm{PDMS}}{}^{2}}{{12(1\;-\;v}_{\mathrm{PDMS}}{}^{2})}\;+\;\frac{Y_{\mathrm{Parylene}}h_{\mathrm{Parylene}}{}^{2}}{{12(1\;-\;v}_{\mathrm{Parylene}}{}^{2})}.$$

where, D is the stiffness of a polymer film with a thickness, h, Young′s modulus, Y ($Y_{\mathrm{PDMS}}$ = 2.97 MPa [41], $Y_{\mathrm{Parylene}}$ = 2.75 GPa [42]), and the Poisson′s ratio, v ($v_{\mathrm{PDMS}}$ = 0.5 [41], $v_{\mathrm{Parylene}}$ = 0.4 [43]). As shown in Figure 6, the total stiffness ($D_{tot}$) trends to increase with increasing the thickness of the parylene layer, and the total stiffness of the hybrid structure with two 2 μm-parylene layers and a 200 μm-PDMS layer is 17.6 × 10^−3^ Pa·m^2^, which is 33% greater than the PDMS stiffness ($D_{\mathrm{PDMS}}$).

### 3.2. Air Pocket Entrapment

Previous studies have reported that air pocket entrapment can occur depending on the aspect ratio and contact angle when a microstructure is present in the polymer layer [22,23,24]. The air pocket entrapment on the electrode site can cause a decrease in the signal-to-noise ratio (SNR) when recording neural signals [15]. However, polymer coating, which is widely used for insulation and protection of circuit boards and wires in the typical manufacturing process of planar electrodes, results in the electrode site being present in the concave microstructure due to its thickness. Furthermore, surfaces with microstructures are super hydrophobic, and this phenomenon can also occur in nature [44].

**Figure 7:**
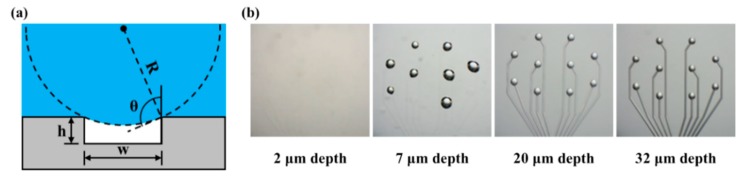
The experiment of air pocket entrapment. (**a**) Schematic of air pocket entrapment and variable descriptions (R: water radius, θ: contact angle, *w*: trench width, *h*: trench depth). (**b**) Experimental results of air pocket entrapment according to depth of concave structure. Some images were adapted and modified from a doctoral dissertation [25,26].

Schrauth [24] theoretically proved the conditions under which air pocket entrapment occurs depending on the aspect ratio and the contact angle of the PDMS concave microstructure. Based on this theory, we also proved mathematically the conditions under which air pocket entrapment occurs. A concave structure with height *h*, width *w*, and water contact angle θ can cause air pocket entrapment on soaking in water. It is assumed that gravity is negligible because the concave structure is sufficiently small. Assuming that the interface between the air pocket and the water has a radius R as shown in Figure 7a, the condition in which air pocket entrapment does not occur is when d is less than h. Then the equation can be derived as follows:

(2) $$\frac{h}{w}\;<\;\frac{1\;-\;\sin\theta }{-2\cos\theta }.$$

According to the above Equation (2), air pocket entrapment occurs when the aspect ratio of the concave structure becomes larger than the k value determined by the contact angle θ. Since the water contact angle θ of PDMS is known to be 99.2 ± 2.27° [45], the aspect ratio k is approximately 0.04. Therefore, air pocket entrapment occurs when the 100 μm-width concave structure has a depth of more than 4 μm. Similar to the results derived from the above equation, the 100 μm-width concave structure placed into water at 25 °C with a contact angle of 45° has no air pocket entrapment at a depth of 2 μm but air pocket entrapment at a depth of more than 7 μm. The number of air pocket entrapments is 0, 8, 10, and 10 at depths of 2, 7, 20, and 32 μm, respectively (Figure 7b). Therefore, in this paper, we proposed the fabrication of an MEA which has electrode sites on the convex microstructures instead of on the concave microstructures as a method to prevent air pocket entrapment.

### 3.3. Animal Experiments

**Figure 8:**
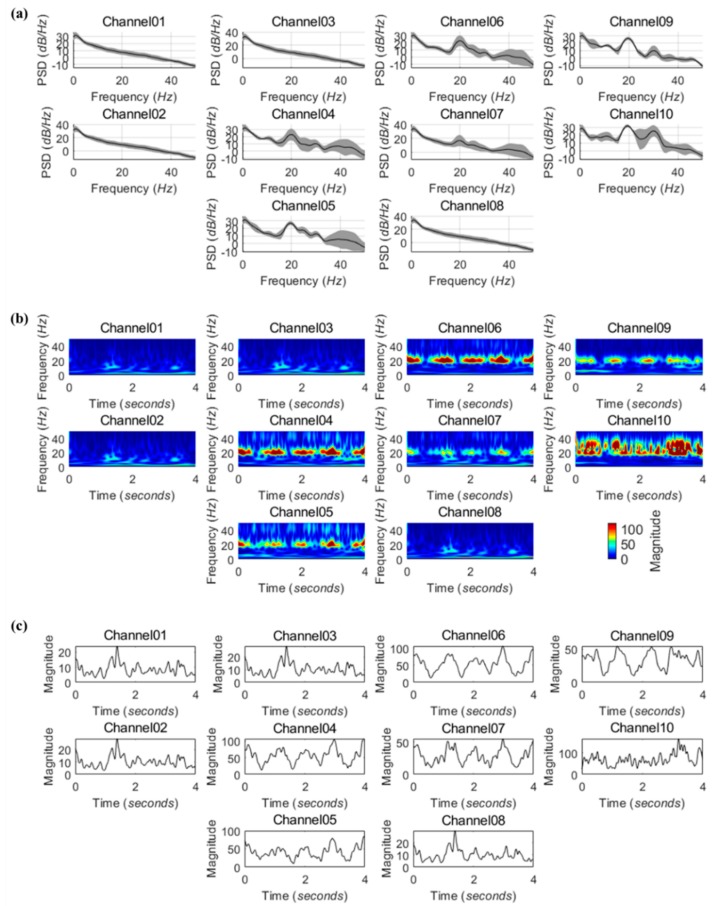
Frequency components of ECoG signals recorded from a rat olfactory bulb via 10 channels of MEA during respiration. (**a**) Averaged power spectral density estimates derived from 20 epochs. Shaded areas indicate the standard deviation; (**b**) Representative time-frequency distributions extracted from a single epoch; (**c**) Time courses of spectral power in the beta frequency band during a single epoch.

In this study, we investigated the feasibility of the MEA by measuring the change of ECoG signals from rat olfactory bulb during respiration with odor-free air. The temporal patterns of the ECoG signals were similar across all animals, as previous studies have shown, and the results showed that the beta frequency band (13–30 Hz) is associated with respiration [46,47,48]. Figure 8 illustrates the results of frequency component analysis of the ECoG signals recorded through 10 channels of MEA during respiration.

## 4. Conclusions

Microstructures made of hydrophobic polymers such as PDMS can cause air pocket entrapment that interferes with the conformal contact of the electrodes, depending on the aspect ratio and the contact angle. We proposed a fabrication method that forms convex microstructures using PDMS dry etching and underexposure photolithography processes [49]. However, problems such as very low etch rate, residue generation, and substrate damage are prone to occur in the PDMS dry etching process. Further, in the underexposed photolithography process that creates undercut structures of negative photoresist, patterns may be created irregularly because the exposure light cannot reach the wafer uniformly. Therefore, in this paper, a fabrication method of an MEA with hybrid convex microstructures protruding about 20 μm from the surface through a photolithography and molding process, instead of the PDMS dry etching and the underexposed photolithography processes, was proposed for conformal contact with the curved surface of the brain. The fabricated MEA has four arms with a length of 1.0–2.0 mm and a width of 600 μm, and ten electrode sites that protrude about 20 μm from the surface. The hybrid convex microstructures consist of a PDMS layer to minimize physical damage due to mechanical mismatch with the neural tissue, two parylene layers to compensate for the disadvantages of the fabrication process for PDMS-based MEAs, and a metal layer to transmit the ECoG signal. The feasibility of the MEA with hybrid convex microstructures was examined by measuring ECoG signals from a rat olfactory bulb during respiration with odor-free air. The results of time-frequency analysis show that the beta frequency band (13–30 Hz) is associated with respiration as in previous studies [46,47,48]. It is expected that this will be applied to various neural recording and stimulation experiments of retina or muscle in addition to the brain with these convex-shaped electrodes.
